# The impact of elder abuse training on subacute health providers and older adults: study protocol for a randomized control trial

**DOI:** 10.1186/s13063-024-08160-3

**Published:** 2024-05-22

**Authors:** Marina G. Cavuoto, Simona Markusevska, Catriona Stevens, Patricia Reyes, Gianna Renshaw, Micah D. J. Peters, Briony Dow, Peter Feldman, Andrew Gilbert, Elizabeth Manias, Duncan Mortimer, Joanne Enticott, Claudia Cooper, Josefine Antoniades, Brenda Appleton, Sigrid Nakrem, Meghan O’Brien, Joan Ostaszkiewicz, Marion Eckert, Cheryl Durston, Bianca Brijnath

**Affiliations:** 1 Social Gerontology, National Ageing Research Institute, PO Box 2127, Parkville, VIC 3050 Australia; 2 Turner Institute for Brain and Mental Health, Monash University, Clayton, VIC Australia; 3 Edith Cowan University, Mount Lawley, WA Australia; 4. Vincent’s Health Sydney, Darlinghurst, NSW Australia; 5 University of New South Wales, Kensington, NSW Australia; 6 Uniting War Memorial Hospital, Waverley, NSW Australia; 7 Sir Charles Gairdner Osborne Park Health Care Group, Stirling and Nedlands, WA Australia; 8 Rosemary Bryant AO Research Centre, Clinical Health Science, University of South Australia, Adelaide, South Australia Australia; 9 Australian Nursing and Midwifery Federation (Federal Office), Melbourne, VIC Australia; 10 Adelaide Nursing School, Faculty of Health and Medical Sciences, University of Adelaide, Adelaide, South Australia Australia; 11 Health Evidence Synthesis, Recommendations, and Impact (HERSI), School of Public Health, University of Adelaide, Adelaide, South Australia Australia; 12 The University of Melbourne, Parkville, VIC Australia; 13 Deakin University, Waurn Ponds, Victoria Australia; 14 La Trobe University, Bundoora, VIC Australia; 15 Monash Centre for Health Research and Implementation, Monash University, Clayton, VIC Australia; 16 Department of Medicine, The Royal Melbourne Hospital, The University of Melbourne, Melbourne, VIC Australia; 17 Centre for Health Economics, Monash University, Caulfield East, VIC Australia; 18 Wolfson Institute of Population Health, Queen Mary University of London, London, UK; 19 East London NHS Foundation Trust, London, UK; 20 Global and Women’s Health, School of Public Health and Preventive Medicine, Monash University, Melbourne, VIC Australia; 21 Department of General Practice, University of Melbourne, Melbourne, VIC Australia; 22 School of Media, Creative Arts and Social Inquiry, Curtin University, Bentley, WA Australia; 23 Melbourne, Australia; 24 Norwegian University of Science and Technology, Trondheim, Norway; 25 Peninsula Health, Frankston, VIC Australia; 26 Health and Innovation Transformation Centre, Federation University, Ballarat, VIC Australia; 27 College of Nursing & Health Sciences, Flinders University, Adelaide, South Australia Australia; 28 Warrnambool, Australia; 29 The School of Social Sciences, The University of Western Australia, Perth, WA Australia

**Keywords:** Elder abuse, Training, Older people, Subacute, Intervention, Co-design, Pragmatic trial

## Abstract

**Background:**

Elder abuse often goes unreported and undetected. Older people may be ashamed, fearful, or otherwise reticent to disclose abuse, and many health providers are not confident in asking about it. In the *No More Shame* study, we will evaluate a co-designed, multi-component intervention that aims to improve health providers’ recognition, response, and referral of elder abuse.

**Methods:**

This is a single-blinded, pragmatic, cluster randomised controlled trial. Ten subacute hospital sites (i.e. clusters) across Australia will be allocated 1:1, stratified by state to a multi-component intervention comprising a training programme for health providers, implementation of a screening tool and use of site champions, or no additional training or support. Outcomes will be collected at baseline, 4 and 9 months. Our co-primary outcomes are change in health providers’ knowledge of responding to elder abuse and older people’s sense of safety and quality of life. We will include all inpatients at participating sites, aged 65 + (or aged 50 + if Aboriginal or Torres Strait Islander), who are able to provide informed consent and all unit staff who provide direct care to older people; a sample size of at least 92 health providers and 612 older people will provide sufficient power for primary analyses.

**Discussion:**

This will be one of the first trials in the world to evaluate a multi-component elder abuse intervention. If successful, it will provide the most robust evidence base to date for health providers to draw on to create a safe environment for reporting, response, and referral.

**Trial registration:**

ANZCTR, ACTRN12623000676617p. Registered 22 June 2023.

**Supplementary Information:**

The online version contains supplementary material available at 10.1186/s13063-024-08160-3.

## Key points

- Underreporting of elder abuse is significant due to barriers to disclosure.

- Health providers may not feel confident in asking about or responding to elder abuse.

- Hospital-based health providers are in a unique position to detect and respond to elder abuse.

- We report our protocol for *No More Shame*, a pragmatic cluster randomised control trial that aims to train health providers to recognise and respond to elder abuse.

- Our outcomes include health providers’ knowledge and management of elder abuse, older people’s quality of life and sense of safety, and rates of elder abuse detection and referrals at hospital sites.

**Supplementary Information:**

The online version contains supplementary material available at 10.1186/s13063-024-08160-3.

## Introduction

Elder abuse refers to harm caused to an older person by a single or repeated act within a relationship of trust. It can include financial, physical, psychological, sexual abuse, and neglect [1, 2] and is commonly perpetrated by adult children [3]. Elder abuse is associated with increased morbidity and mortality including higher rates of depression, anxiety, fear, stress, substance dependence, social isolation, poorer physical health, and suicide [4–6]. Community prevalence is around 15% [1], although rates of underreporting are high [7].

Specialised health, aged care, community, and legal services directly involved in care for older people experiencing abuse hold valuable expertise about recognising and responding to elder abuse [8]. However, many frontline health providers do not recognise elder abuse, do not report suspected cases, and often lack the time, confidence, and knowledge to respond [9, 10]. Additionally, ageist attitudes amongst health providers may impede older people’s care [11]. Ageism is a recognised driver of elder abuse as it perpetuates notions that older people are dependent, an economic burden, and have less relevance, which in turn lead to greater tolerance of abuse [12].

Reviews [13, 14] consistently identify the most effective interventions to stop and prevent further occurrence of elder abuse include educating health providers and involving multidisciplinary services. The few randomised control trials (RCTs) in this area mostly focus on one of these interventions, demonstrating that training health providers can improve awareness and knowledge elder abuse response [15, 16] and improve how student practitioners detect financial elder abuse [17]. Evidence regarding the impact of training on health providers’ attitudes, detection, and reporting rates within health services is sparse [10, 18, 19].

We aim to test whether a multimodal intervention combining health provider training, provision of a screening tool, and a site champion improves health providers’ knowledge and management of abuse and older people’s sense of safety and quality of life over 9 months, compared to no additional training or support in Australian subacute hospitals.

Subacute hospitals provide multidisciplinary care to optimise functioning and quality of life, including rehabilitation, geriatric evaluation and management, psychogeriatric care [20], and often involve longer stays to manage complex health conditions before discharge. This provides an important opportunity for clinicians to build trust [8]. This may be the only time an older person experiencing abuse leaves their home for a prolonged period, spending time away from the perpetrator. Indeed, the abuse itself may precipitate or prolong their hospital stay [21].

### Aims and hypotheses

*Our primary aim *is to test the hypothesis that health providers in the intervention arm will show greater knowledge of responding to elder abuse at 4 months relative to the control arm and that older adults in the intervention arm will show greater quality of life at 4 months than those in the control arm.

## Methods

### Design and theory

**Fig. 1:**
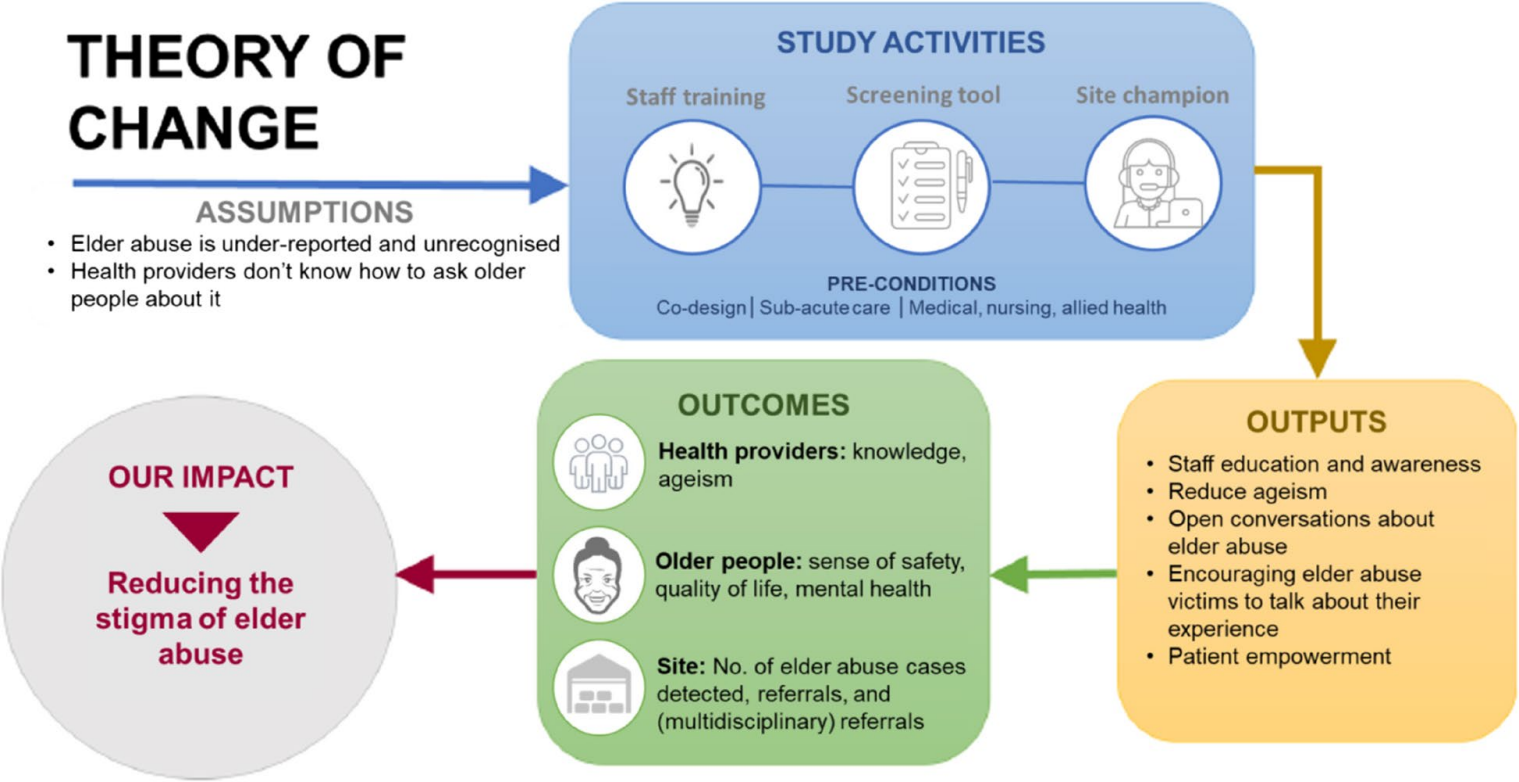
Our intervention is guided the Theory of Change [22]. We conceptualise stigma as an interpersonal, social phenomenon. Our intervention reduces this stigma by creating a safe and inclusive relationship between health providers and older people. Through staff training, educating and modelling; implementation of a screening tool; and champions to sustain processes; we create, measure, and sustain practice change

This is a pragmatic, cluster RCT. The intervention is guided by the Theory of Change [22], which seeks to create a safe and inclusive relationship between health providers and older people (see Fig. 1). We use the SPIRIT reporting guidelines (see Additional file 1) [23].

### Participants and setting

#### Sites

Subacute hospitals providing inpatient care in New South Wales, South Australia, Victoria, and Western Australia were included.

#### Health providers

We aim to recruit inpatient subacute clinical staff providing direct care to older people, including medical, nursing, or allied health. We will exclude staff who do not expect to be working at the site for at least 12 months post-baseline or who participated in co-designing the training.

#### Older people

We will include all inpatients at participating sites, aged 65 + (or aged 50 + if Aboriginal or Torres Strait Islander), who are willing to participate and individually capable of providing informed consent.

Exclusion criteria for older people are (i) receiving palliative care and (ii) lack of decision-making capacity.

### Intervention

This intervention comprises 3 components:

(i) *Training*: Health provider participants will be asked to complete training via an online Learning Management System (Acorn) hosted by the National Ageing Research Institute (NARI). The training takes ~ 60 min to complete and is self-paced, and participants will have access to it over a 2-month period. It has four modules on knowledge of abuse, drivers of abuse, screening for abuse (including how to administer the Australian Elder Abuse Screening Instrument [AUSI]) [24], and management of elder abuse (further details available through the online trial registry anzctr.org.au, registration #ACTRN12623000676617p). This training was co-designed through workshops, interviews, and user-testing with health providers, family carers, older people, and elder abuse survivors. Site champions will promote completion of the training, with the aim of at least 60% of eligible health providers on participating wards completing the training over a 2-month period.
(ii) *Screening tool*: Intervention sites will be provided with the AUSI screening tool, which has content validity [24], and has been demonstrated to increase staff confidence in screening for abuse and the proportion of cases of suspected abuse that provoked multidisciplinary responses [25]. Following the training, health providers will be asked to screen all patients for elder abuse and to manage detected cases as per usual practice, including referrals to outside agencies, but with support from the site champion where required.
(iii) *Site champion*: Each site will recruit an on-site clinician (senior social worker or other staff with commensurate experience) as a site champion, who will support other hospital staff in screening and managing elder abuse. They will be trained by the research team and work 1 day per week supporting the intervention. Site champions will encourage uptake of the training and the AUSI and regularly contact staff to assess concerns and/or challenges encountered with screening. Site champions will be contacted every 2–3 weeks via email/phone by the research team to proactively support them and build a national peer-support network [26].

### Control

The control arm will be waitlisted and given access to the training after 9 months. Control arm sites will also recruit site champions, who will be trained and supported in data collection. They will encourage completion of the outcome measures for health providers, recruit older people and collect data, and collect deidentified site data but will not provide further input beyond their normal clinical role.

### Outcome assessments

All health provider participants will complete baseline assessment prior to site randomisation. Outcomes will be assessed at baseline (t0), 4 months follow-up (t1), and 9 months follow-up (t2) (see Table 1).

#### Sites

Site champions will obtain summary statistics routinely collected by hospitals such as general site characteristics (size of workplace, location, subacute services offered) and characteristics of patients admitted to participating wards (age, gender, country of birth, English proficiency, reason for subacute stay). They will also ascertain the number of eligible health providers to enable the calculation of the percentage who complete the training. Elder abuse detection and referral data will be collected from the 9 months preceding the trial to the end of the trial (i.e. at completion of 9-month outcomes). Specifically, site champions will screen medical records of all inpatients on participating wards during that time to ascertain (i) number of records screened, (ii) number of cases that identified elder abuse and the types, (iii) referrals made to social work, and (iv) the frequency of interventions and referrals (e.g. legal referral, safety planning) using a standardised template that will be provided. In the intervention arm, the number of times the AUSI screening tool is administered and not administered will also be collected; however, the responses on the AUSI will not be collected.

#### Health providers

Health providers will complete a demographic survey (age, gender, profession, years of experience in profession, and whether they have completed elder abuse training prior to the trial). They will also complete the adapted Knowledge and Management of Abuse (KAMA) [27] (primary outcome) adapted for an Australian subacute care setting, the Australian adaptation of the Caregiver Scenario Questionnaire (CSQ) [28, 29], and Carolina Opinions on Care of Older Adults (COCOA) survey [30] (secondary outcomes) via online survey in REDCap.

#### Older people

Older people will complete a demographic survey (age, gender, education, postcode, country of birth, language/s spoken, English language skills, First Nations status, reason for admission and underlying health risk factors for elder abuse (e.g. dementia, mobility), the Adult Social Care Outcomes Toolkit (ASCOT) [31] (primary outcome), the Short Form Survey-12 (SF-12v2) [32], and an adapted Resource Utilisation in Dementia lite (a-RUD-lite) (secondary outcomes) via online/paper/telephone survey. Site champions will support older people where required and record the older person’s length of admission once discharged.

**Table 1:** Procedure timeline

**Procedures**	**Assessment/procedure**	**Screening**	**Baseline**	**4-month follow-up**	**9-month follow-up**
	**Health providers**				
	** Informed consent**	**x**			
	** Demographic Information**		**x**		
	** a-KAMA questionnaire**		**x**	**x**	**x**
	** CSQ**		**x**	**x**	**x**
	** COCOA questionnaire**		**x**	**x**	**x**
	**Older adults**				
	** Informed consent**	**x**			
	** Demographic Information**		**x**		
	** ASCOT questionnaire**		**x**	**x**	**x**
	** SF-12v2 questionnaire**		**x**	**x**	**x**
	** a-RUD-lite questionnaire**		**x**	**x**	**x**
	** Has patient attended another hospital in trial during study period**		**x**	**x**	**x**
	**Administrative/site data**	**9 months prior to Baseline**	**Baseline**	**Baseline to 4 months**	**4-months to 9 months**
	** Elder abuse cases**	**x**	**x**	**x**	**x**
	** Elder abuse referrals**	**x**	**x**	**x**	**x**
	** Nature of referral**	**x**	**x**	**x**	**x**
	** Number of eligible clinicians (total and by profession)**		**x**	**x**	
	** Number of clinicians who have completed the training (total and by profession)**		**x**	**x**	
	** Number of times the screening tool was administered/not administered**		**x**	**x**	**x**

#### Process evaluation

We will conduct a multi-method process evaluation on how the intervention works in practice. Training completion rates will be monitored using the NARI Learning Management System. In addition to monitoring uptake of the screening tool, a researcher will visit each intervention site for 2–3 days approximately 3 months after implementation of the screening tool. We will observe how the screening and referrals are conducted and interview site champions and 20–25 health providers across the sites. This evaluation will mobilise WHO guidance for scaling up health service innovations [33] as we seek to understand the following: intervention uptake, potential facilitators/barriers, and solutions; whether the essential features of the intervention are being adhered to in practice (and if not, what remedial action can or should be taken); and whether the intervention improved elder abuse detection and response and impacted practice and provider satisfaction.

### Sample size

As health providers are not anticipated to be systemically different based on location (clusters), we have not accounted for ICC for health providers; therefore, we calculated that 60 health providers would be sufficient to detect a medium effect (Cohen’s *d* of 0.5) on the KAMA at a power of 80% and a significance level of 1%. This difference is similar to that reported in our previous research, and the sample size calculation uses a standard deviation of 4.8 as previously reported [34]. We calculated that we need 10 clusters (5 intervention, 5 control) with 40 older people in each cluster to detect a medium effect on the ASCOT at a power of 80% and a significance level of 5%. As responses to elder abuse vary across jurisdictions, an ICC of 0.1 is assumed for older people with the standard deviation of 0.2 reported by others [35], calculated using the Shiny Calculator for Sample size for Cluster Randomised Trials [36]. Assuming 53% refusal rate to participate/unable to consent as observed in our group’s prior work in the UK [37, 38], the total sample size is 92 health providers and 612 older people (~ 60 older people/site).

### Recruitment

Site champions will recruit health providers and older people in intervention and control arms. They will promote the study to subacute staff, during staff meetings, through posters and emails targeting relevant staff. A QR code will link to further information about the study. For the 4- and 9-month follow-up, site champions will remind staff up to 3 times verbally or by email.

Site champions will prospectively recruit all older patients who meet eligibility criteria, as identified through staff meetings, medical files, and discussion with patients. They will provide each eligible participant with an invitation letter and PICF. Older people can choose to complete paper-based surveys with a reply-paid envelope provided or a text message or email link with the necessary information, as we have successfully used in prior research [39]. Older people will be recruited once the health providers in the intervention arm have completed the training and use of the screening tool has been implemented at the site; therefore, the baseline, 4-month, and 9-month time points for older people will be slightly later.

Based on stakeholder consensus, we aim for 60% of eligible staff to complete the training prior to commencing the implementation of the screening tool to maximise patient exposure. It is possible that an older adult participant in the intervention arm will receive clinical care from someone who has not completed the training. Thus, we will also capture the ‘dose’ for each site as a percentage of eligible staff who have completed the training.

### Group allocation

Stratified randomisation will allocate clusters (subacute sites) to either intervention or wait list control using a minimisation procedure. State (VIC, NSW, SA, WA) is a stratification factor. This is a single-blinded study where the statistician and outcome assessors will be blinded to allocation. Randomisation will occur following completion of baseline data collection for health providers. An independent biostatistician who is not a member of the research team will generate the allocation sequence. It is not possible to blind participants to group allocation when collecting the post-intervention scores. During the assessment period, we will ask health provider participants not to discuss the study outside their site.

### Data collection and management

For health providers, outcome questionnaires will take approximately 30–40 min to complete through the NARI-hosted REDCap database. For older people, questionnaires will take approximately 40 min. Outcomes will be entered into an electronic database by the research team and stored on a secure server. Participants will not be identifiable from any data that is published or otherwise publicly released. As the intervention is not a medical intervention, a data monitoring committee was not required by the Human Research Ethics Committee. Older adult participant data will be screened by site champions for any responses that indicate a concern requiring clinical follow-up (e.g. low mood) which will be undertaken by the site champion through their normal role as a senior social worker at the site, according to usual practice.

### Promoting retention and follow-up

Intervention and control sites will receive a gift hamper at each follow-up to encourage survey completion. Survey reminder notices will be distributed to all sites’ staff with a QR code for survey completion; at each of the three assessment points, those completing the survey will enter a draw for a $250 gift card. A poster benchmarking the site’s progress in survey completion against other sites (de-identified) and study promotion materials will also be placed in staff lunchrooms.

### Analysis

#### Primary and secondary RCT outcomes

The primary analysis will be performed according to intention to treat, including all clusters and participants in the allocated groups. Between-group difference for both primary outcomes (health provider and older people) will be analysed using mixed effects regression models with a fixed effect for the intervention group and random effect for participants to account for repeated measures. Cluster site will also be added as a random effect. Due to the likelihood of differences across sites, a number of baseline covariates will be controlled for in the analyses (as listed in Table 2).

**Table 2:** List of covariates to be used in analysis and source

	*Health provider outcomes*	*Older adult outcomes*
*Self-reported*	Profession, age, gender, years in the role	Age, sex, ethnicity, location, educational and economic attainments, country of birth, English proficiency, reason for subacute stay, and admission to any other hospital in the trial
*Reported by site champion based on data routinely collected by hospitals, or case records*	General characteristics of the site (size of workplace, location, subacute services offered), average characteristics of older patient cohorts admitted to the participating wards (age, gender, country of birth, English proficiency, reason for subacute stay), and elder abuse cases (number of suspected elder abuse cases p/month, number, and types of referral) to enable the evaluation and statistical control of differences between sites	Length of hospital admission, length of admission on participating ward; number of eligible clinicians (total and by profession), and number of clinicians who have completed the training (total and by profession) to provide a measure of ‘dose’

We will similarly analyse secondary outcomes. Continuous outcomes will be analysed using mixed effects linear regression models and adjustment for baseline values. Statistics will be reported with their respective 95% confidence intervals and *P* values. Per-protocol analyses will also be performed, omitting participants with predefined protocol deviations (e.g. allocated intervention not received, violated inclusion/exclusion criteria). The trial results will be reported in line with the CONSORT extension for cluster trials. As this is a low-risk, non-medical intervention, no interim analyses will be conducted, and no stopping guidelines were developed for ceasing the trial early. Similarly, as adverse events reporting is only required for medical interventions, we do not report these.

#### Fidelity and acceptability

Fidelity/adherence data will be derived from training completion rates and the process evaluation. Acceptability of the intervention is pre-specified as > 70% of health provider participants rating the intervention ‘completely acceptable’ at 9 months [40].

#### Process analysis

Qualitative data collected during the process analysis interviews and observations will be thematically analysed using an inductive and reflexive coding approach to iteratively revise and define semantic themes [41] and managed through NVivo. Revisions to the Theory of Change will be iteratively discussed by the research team, and a final determination of the Theory of Change will be reached by team consensus.

#### Economic analysis

We will undertake a trial-based analysis to describe the additional costs (savings) and consequences arising from our intervention as compared to our usual care control condition. The cost-effectiveness analysis will capture two types of costs: (1) participant direct medical (primary and allied health care, medications, acute and subacute admissions) and social care costs (paid and unpaid home care and transitions to residential care) estimated from an adapted RUD-lite [42] plus supplementary self-report at baseline, 4 months, and 9 months and (2) costs associated with implementing the intervention and control conditions estimated from administrative and fidelity data. We will relate cost per participant to patient-level measures of safety, quality of life, and mental health. In line with the main analysis, the primary outcome for the economic evaluation will be social care quality adjusted life-years (QALYs) to final follow-up calculated based on ASCOT scores [31, 43] at baseline, 4 months, and 9 months. The secondary outcome for the economic evaluation will be health QALYs to final follow-up calculated based on SF12v2-based SF6D scores [44] at baseline, 4-month, and 9-month data. Following recent recommendations [45], results will be expressed as (i) cost per ASCOT-based social care QALY and (ii) cost per SF6D-based health QALY. We will summarise sampling error and decision uncertainty using the bootstrap acceptability method to calculate confidence intervals and generate cost-effectiveness acceptability curves [46].

### Oversight and monitoring

A 6 monthly project Stakeholder Advisory Group will provide oversight and advice of the project. This will include those with lived experience of elder abuse, older people, family carers, health providers, service providers, educators, and advocacy groups experienced in elder abuse responses.

This project has been ethically reviewed and approved by Austin Health Human Research Ethics Office through the National Mutual Acceptance Scheme with governance approval to be provided by each hospital site. Any changes to the protocol will be submitted to the Austin Health Human Research Ethics Office and all site Human Research Ethics Offices and will be updated on the ANZCTR where necessary.

## Discussion

There is a need for high-quality trials with adequate statistical power and appropriate study characteristics to determine what is effective in preventing or reducing elder abuse [47]. Addressing this call, this will be one of the few multi-component elder abuse RCTs in the world.

Our pragmatic approach aims to improve elder abuse knowledge, screening, and response in as many subacute health providers as possible rather than to provide extensive, specialised, or advanced training to fewer health providers who are under increasing demands due to pressures on the health care system. Our rationale is that upskilling more healthcare providers will result in increased detection of elder abuse and better response and management with beneficial flow-on effects to older people.

As both the training and screening tool have been co-designed, we expect high rates of acceptability amongst health providers. However, our multi-method process evaluation will allow us to determine what refinements are needed prior to national implementation.

### Limitations

We will exclude patients who receive subacute care at home, despite this being an increasingly popular model of care. This minimises the risk of screening in the presence of a perpetrator, which would reduce the likelihood of disclosing abuse and may pose risks to the older person and/or care staff.

Second, we will exclude older adults without the capacity to consent to research participation to avoid seeking proxy consent from a substitute decision maker, which could include a perpetrator of abuse.

Finally, the recruitment of older people by site champions after randomisation introduces a risk of bias as site champions will know whether they are in the intervention or control arm. This common problem with cluster RCTs will be managed in the analytic strategy (e.g. through difference-in-differences techniques).

### Dissemination policy: trial results

Trial results will be disseminated by publication in scientific journals and conferences. Following publication, the findings will also be promoted through stakeholder and industry newsletters, social media, and a national stakeholder forum at completion of the trial and through networks of international researchers involved in the study. Results will be disseminated regardless of the direction or magnitude of the effect.

### Dissemination policy: authorship

Authorship will be based on substantive contributions to the design, conduct, interpretation, and reporting and will include the investigator team, core research team, and consumers involved in investigator meetings.

Site principal investigators will be granted authorship for their contributions to the trial. Site champions and the Stakeholder Advisory Group will be included in the acknowledgments. Professional writers will not be used.

### Dissemination policy: reproducible research

The trial protocol and anonymised participant-level dataset will be made available upon request.

### Trial status

Protocol version 3, 11 September 2023. Recruitment has commenced. The approximate date when recruitment will be completed is 29 August 2025.

## Conclusions

Elder abuse often goes unreported and undetected. Older people may not feel comfortable to disclose it, and health providers may not know how to screen for or respond to it. Our work is a valuable first step to improving the health provider response to addressing this pernicious social issue.

### Supplementary Information

Additional file 1. SPIRIT Checklist for *Trials*.

## Data Availability

All members of the research team will have access to the final dataset. Access to the de-identified datasets will be available from the project lead investigator on request.

## References

[1] Qu L, Kaspiew R, Carson R, Roopani D, De Maio J, Harvey J, Horsfall B. National Elder Abuse Prevalence Study: Final Report. (Research Report). Melbourne: Australian Institute of Family Studies; 2021.

[2] World Health Organization. Abuse of older people. 2008. Available from: https://www.who.int/news-room/fact-sheets/detail/abuse-of-older-people.

[3] Brijnath B, Gartoulla P, Joosten M, Feldman P, Temple J, Dow B (2021). A 7-year trend analysis of the types, characteristics, risk factors, and outcomes of elder abuse in community settings. J Elder Abuse Negl.

[4] Dong X, Simon M, Mendes de Leon C, Fulmer T, Beck T, Hebert L (2009). Elder self-neglect and abuse and mortality risk in a community-dwelling population. JAMA.

[5] World Health Organization (WHO) (2011). European report on preventing elder maltreatment.

[6] Dong X, Chen R, Chang E-S, Simon M (2013). Elder abuse and psychological well-being: a systematic review and implications for research and policy-A mini review. Gerontology.

[7] Dow B, Brijnath B (2019). Elder abuse: context, concepts and challenges. Australia’s welfare 2019 data insights: Australia’s welfare series no 14 Cat No AUS 226.

[8] Brijnath B, Gahan L, Gaffy E, Dow B (2020). “Build rapport, otherwise no screening tools in the world are going to help”: frontline service providers’ views on current screening tools for elder abuse. Gerontologist.

[9] Dow B, Gaffy E, Hwang K. Elder abuse community action plan for Victoria. National Ageing Research Institute; 2018.

[10] Cooper C, Selwood A, Livingston G (2009). Knowledge, detection, and reporting of abuse by health and social care professionals: a systematic review. Am J Geriatr Psychiatry.

[11] Gallo V (2019). Ageism in nursing education: a review of the literature. Teach Learn Nurs.

[12] Phelan A, Ayalon L (2020). The intersection of ageism and elder abuse. Advances in elder abuse research: practice, legislation and policy, international perspectives on aging.

[13] Owusu-Addo E, O’Halloran K, Birjnath B, Dow B. Primary prevention interventions for elder abuse: A systematic review. (Research Report). Prepared for Respect Victoria on behalf of National Ageing Research Institute. 2020.

[14] Lachs MS, Pillemer KA (2015). Elder abuse. N Engl J Med.

[15] Richardson B, Kitchen G, Livingston G (2002). The effect of education on knowledge and management of elder abuse: a randomized controlled trial. Age Ageing.

[16] Mohd Mydin FH, Wan Yuen C, Othman S, Mohd Hairi NN, Mohd Hairi F, Ali Z, et al. Evaluating the effectiveness of I-NEED program: improving nurses’ detection and management of elder abuse and neglect—a 6-month prospective study. J Interpers Violence. 2022;37(1–2):NP719–41. 10.1177/0886260520918580 32394780

[17] Harries P, Davies M, Gilhooly K, Gilhooly M, Tomlinson C (2014). Educating novice practitioners to detect elder financial abuse: a randomised controlled trial. BMC Med Educ.

[18] Garma CT (2017). Influence of health personnel’s attitudes and knowledge in the detection and reporting of elder abuse: an exploratory systematic review. Psychosoc Interv.

[19] Mohd Mydin FH, Yuen CW, Othman S (2021). The effectiveness of educational intervention in improving primary health-care service providers’ knowledge, identification, and management of elder abuse and neglect: a systematic review. Trauma Violence Abuse.

[20] Australian Institute of Health and Welfare (2014). Australia’s health 2014.

[21] Collins M, Posenelli S, Cleak H, O’Brien M, Braddy L, Donley E (2020). Elder abuse identification by an Australian Health Service: a five-year, social-work audit. Aust Soc Work.

[22] Stein D, Valters C (2012). Understanding theory of change in international development.

[23] Chan A-W, Tetzlaff J, Gøtzsche P, Altman D, Mann H, Berlin J (2013). SPIRIT 2013 explanation and elaboration: guidance for protocols of clinical trials. BMJ.

[24] Gahan L, Gaffy E, Dow B, Brijnath B (2019). Advancing methodologies to increase end-user engagement with complex interventions: the case of co-designing the Australian elder abuse screening instrument (AuSI). J Elder Abuse Negl.

[25] Brijnath B, Gahan L, Dow B, Hickey L, Braddy L, Collins M (2022). When co-design works (sort of): the case of the Australian elder abuse screening instrument. J Elder Abuse Negl.

[26] Hernandez-Tejada MA, Skojec T, Frook G, Steedley M, Davidson TM (2021). Addressing the psychological impact of elder mistreatment: community-based training partnerships and telehealth-delivered interventions. J Elder Abuse Negl.

[27] Richardson B, Kitchen G, Livingston G (2003). Developing the KAMA instrument (knowledge and management of abuse). Age Ageing.

[28] Selwood A, Cooper C, Livingston G (2007). What is elder abuse—who decides?. Int J Geriatr Psychiatry.

[29] Hempton C, Dow B, Cortes-Simonet E, Ellis K, Koch S, LoGiudice D (2011). Contrasting perceptions of health professionals and older people in Australia: what constitutes elder abuse?. Int J Geriatr Psychiatry.

[30] Hollar D, Roberts E, Busby-Whitehead J (2011). COCOA: a new validated instrument to assess medical students’ attitudes towards older adults. Educ Gerontol.

[31] Netten A, Burge P, Malley J, Potoglou D, Towers AM, Brazier J (2012). Outcomes of social care for adults: developing a preference-weighted measure. Health Technol Assess.

[32] Cheak-Zamora NC, Wyrwich KW, McBride TD (2009). Reliability and validity of the SF-12v2 in the medical expenditure panel survey. Qual Life Res.

[33] World Health O (2009). Practical guidance for scaling up health service innovations.

[34] Cooper C, Huzzey L, Livingston G (2012). The effect of an educational intervention on junior doctors’ knowledge and practice in detecting and managing elder abuse. Int Psychogeriatr.

[35] van Leeuwen KM, Bosmans JE, Jansen AP, Hoogendijk EO, van Tulder MW, van der Horst HE (2015). Comparing measurement properties of the EQ-5D-3L, ICECAP-O, and ASCOT in frail older adults. Value Health.

[36] Hemming K, Kasza J, Hooper R, Forbes A, Taljaard M (2020). A tutorial on sample size calculation for multiple-period cluster randomized parallel, cross-over and stepped-wedge trials using the Shiny CRT Calculator. Int J Epidemiol.

[37] Cooper C, Barber J, Griffin M, Rapaport P, Livingston G (2016). Effectiveness of START psychological intervention in reducing abuse by dementia family carers: randomized controlled trial. Int Psychogeriatr.

[38] Livingston G, Barber J, Marston L, Stringer A, Panca M, Hunter R (2019). Clinical and cost-effectiveness of the Managing Agitation and Raising Quality of Life (MARQUE) intervention for agitation in people with dementia in care homes: a single-blind, cluster-randomised controlled trial. Lancet Psychiatry.

[39] Manias E (2012). Complexities of pain assessment and management in hospitalised older people: a qualitative observation and interview study. Int J Nurs Stud.

[40] Perski O, Short CE (2021). Acceptability of digital health interventions: embracing the complexity. Transl Behav Med.

[41] Terry G, Hayfield N, Clarke V, Braun V (2017). Thematic analysis. The SAGE handbook of qualitative research in psychology.

[42] Wimo A, Wetterholm AL, Mastey V, Winbald B. Evaluations of resource utilization and caregiver time in antidementia drug trials- a quantitative battery. In: Wimo A, Jonsson B, Karlsson G, Winbald B, eds. The health economics of dementia. London: John Wiley & Sons; 1998. p. 465–499.

[43] Malley JN, Towers A-M, Netten AP, Brazier JE, Forder JE, Flynn T (2012). An assessment of the construct validity of the ASCOT measure of social care-related quality of life with older people. Health Qual Life Outcomes.

[44] Brazier JE, Roberts J (2004). The estimation of a preference-based measure of health from the SF-12. Med Care.

[45] Bulamu NB, Kaambwa B, Ratcliffe J (2015). A systematic review of instruments for measuring outcomes in economic evaluation within aged care. Health Qual Life Outcomes.

[46] Glick HA, Doshi JA, Sonnad SS, Polsky D (2014). Economic evaluation in clinical trials.

[47] Baker PR, Francis DP, Hairi NN, Othman S, Choo WY (2016). Interventions for preventing abuse in the elderly. Cochrane Database Syst Rev.

